# Macrophage Polarization Related to Crystal Phases of Calcium Phosphate Biomaterials

**DOI:** 10.3390/ijms222011252

**Published:** 2021-10-19

**Authors:** Linghao Xiao, Yukari Shiwaku, Ryo Hamai, Kaori Tsuchiya, Keiichi Sasaki, Osamu Suzuki

**Affiliations:** 1 Division of Craniofacial Function Engineering, Tohoku University Graduate School of Dentistry, Sendai 980-8575, Japan; linghao.xiao.s8@dc.tohoku.ac.jp (L.X.); yukari.shiwaku.a8@tohoku.ac.jp (Y.S.); ryo.hamai.a3@tohoku.ac.jp (R.H.); kaori.tsuchiya.b6@tohoku.ac.jp (K.T.); 2 Division of Advanced Prosthetic Dentistry, Tohoku University Graduate School of Dentistry, Sendai 980-8575, Japan; keiichi.sasaki.e6@tohoku.ac.jp; 3 Liaison Center for Innovative Dentistry, Tohoku University Graduate School of Dentistry, Sendai 980-8575, Japan

**Keywords:** octacalcium phosphate, hydroxyapatite, macrophage polarization, crystal phase, microenvironment

## Abstract

Calcium phosphate (CaP) materials influence macrophage polarization during bone healing. However, the effect of the crystal phase of CaP materials on the immune response of bone remains unclear. In this study, the effect of the crystal phases of CaP materials on the regulation of macrophage polarization was investigated. Human THP-1 cells and mouse RAW 264 cells were cultured with octacalcium phosphate (OCP) and its hydrolyzed form Ca-deficient hydroxyapatite to assess the expression of pro-inflammatory M1 and anti-inflammatory M2 macrophage-related genes. OCP inhibited the excessive inflammatory response and switched macrophages to the anti-inflammatory M2 phenotype, which promoted the expression of the *interleukin 10* (*IL10*) gene. In contrast, HL stimulated an excessive inflammatory response by promoting the expression of pro-inflammatory M1 macrophage-related genes. To observe changes in the microenvironment induced by OCP and HL, inorganic phosphate (Pi) and calcium ion (Ca^2+^) concentrations and pH value in the medium were measured. The expression of the pro-inflammatory M1 macrophage-related genes (tumor necrosis factor alpha (*TNFα*) and interlukin 1beta (*IL1β*)) was closely related to the increase in ion concentration caused by the increase in the CaP dose. Together, these results suggest that the microenvironment caused by the crystal phase of CaP materials may be involved in the immune-regulation capacity of CaP materials.

## 1. Introduction

Immune response is a critical part of tissue restoration and is considered to control the rate and prognosis of the healing process [1]. Bone substitute materials mediate osteogenesis by undergoing the early tissue response, bone regeneration, and bone remodeling phases with the involvement of multiple systems, including the coagulation, immune, and skeletal systems [2]. During this healing process, bone cells and immune cells interact with each other in a microenvironment that includes a variety of molecules in the bone marrow, contributing to osteoimmune functions [3]. A novel concept of osteoimmunomodulation (OIM) has been proposed as a crucial property of bone biomaterials to assess their osteogenesis and osseointegration capacities [4,5]. After the implantation of biomaterials, macrophage activation and polarization to either pro-inflammatory M1 macrophages or anti-inflammatory M2 macrophages, accompanied by the release of immune regulators, result in different prognosis pathways [6]. Both macrophage phenotypes, rather than a specific phenotype, are essential during the osteogenic process [5]. However, limited information is available regarding the osteoimmune response during the implantation of representative bone-substitute materials, such as CaPs. 

Octacalcium Phosphate (OCP), a CaP crystal, is considered a precursor of biological apatite in bones and teeth [7,8]. Compared with some traditional CaP materials, such as hydroxyapatite (HA) and β-tricalcium phosphate (β-TCP), OCP exhibits better osteoconductivity and biodegradability [9,10], and promotes the differentiation of osteoblasts and osteocytes, accompanied by OCP-apatite conversion [11,12,13,14]. Moreover, OCP incorporates calcium ion (Ca^2+^) and releases inorganic phosphate (Pi) ions during its conversion to OCP hydrolyzate (HL), which is a Ca-deficient HA with a crystal morphology similar to that of the original OCP. OCP is also highly biodegradable and can induce osteoclast formation through the expression of the receptor activator of nuclear factor-κB ligand (RANKL) by osteoblasts in a co-culture system [15]. Furthermore, the chemical environment around the OCP affects crystal dissolution and reprecipitation [16] and regulates the coupling factors secreted by osteoclasts [17].

Our previous studies have investigated the effect of OCP on immune responses, particularly macrophages, on histological tissue and culturing cellular levels [16,18,19]. We observed that OCP did not stimulate human monocytes to produce TNFα, suggesting that OCP could be used as a biocompatible material [19]. Compared with HL, OCP moderately increased the immune response by promoting the recruitment of macrophages and stimulating the expression of immune-related cytokines and chemokines, such as chemokine (C-X-C) ligand 2 (CXCL2) and IL6, in the initial inflammatory stage after implantation through ionic dissolution [18,20,21]. Macrophage migration is accompanied by tartrate-resistant acid phosphatase (TRAP)-positive osteoclast formation, which is closely related to the physicochemical milieu regulated by OCP during the entire bone-healing process [18]. However, the induction mechanism of macrophage polarization around OCP and HL crystals during bone formation has not been clarified.

Macrophages are first polarized to the M1 phenotype, which exhibits a pro-inflammatory function of secreting characteristic pro-inflammatory cytokines such as TNFα, IL1β, and IL6, producing an overdose of reactive oxygen species (ROS) [22]. During the inflammatory phase, progenitors and immune cells are recruited and the process of osteoclastogenesis is stimulated, making this phase indispensable [23]. Nevertheless, an effective and timely switch from the M1 phenotype to the anti-inflammatory M2 phenotype determines bone regeneration [5]. Macrophages polarize to alternatively activate M2 macrophages and release anti-inflammatory cytokines, such as IL10, bone morphogenetic protein 2 (BMP2), and vascular endothelia growth factor (VEGF), to provide a bone-regeneration environment and regulate osteogenic differentiation. Physiochemical characteristics of biomaterials, such as surface properties, particle size, porosity, and pore size, contribute to osteogenesis and might be involved in the regulation of the OIM properties and the release of ions [5].

In the present study, we investigated the effect of the crystal phase of CaP materials on the regulation of macrophage polarization. Synthetic OCP was hydrolyzed to obtain Ca-deficient HA with a similar plate-like crystal shape and similar granule size but different crystalline structures. Thus, we compared the immunomodulatory capacities of the two CaP materials. The effect of the crystal phase of CaPs on immunomodulation was demonstrated via changes in macrophage behaviors.

## 2. Results

### 2.1. Identification of the THP-1 Derived M1 and M2 Macrophages

**Figure 1:**
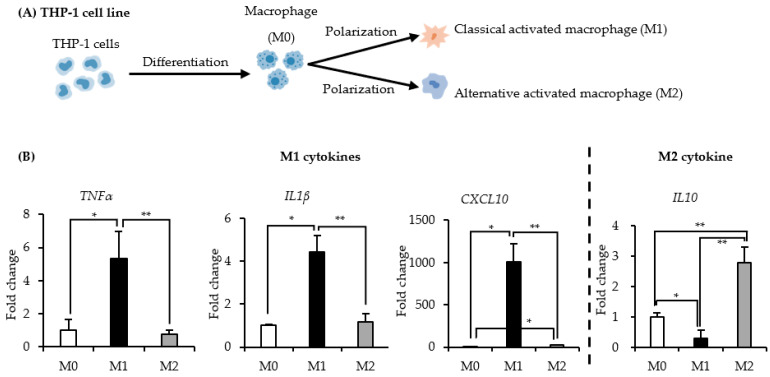
Human monocyte THP-1 cell differentiation and polarization. (**A**) Human monocyte THP-1 cell differentiation and polarization model diagram. THP-1 cells were firstly differentiated to M0 macrophages, and then induced to M1 or M2 macrophages. (**B**) The expression of M1 (*TNFα*, *IL1β*, and *CXCL10*) and M2 (*IL10*) macrophage related cytokines by qPCR. (*n* = 3). ** *p* < 0.01, * *p* < 0.05.

Human THP-1 cells were differentiated and then polarized to M1 and M2 macrophages, as shown in Figure 1A. Human THP-1 monocytes were observed to adhere to the substrate of the culture plate after treatment with 100 ng/mL of phorbol-12 myristate-13 acetate (PMA), which indicated monocyte-to-macrophage differentiation. The expression of M1 macrophage-related cytokines or M2 macrophage-related cytokine was significantly higher in macrophages incubated with lipopolysaccharides (LPS) and interferon gamma (IFNγ) for M1 polarization or IL4 and IL13 for M2 polarization (Figure 1B).

### 2.2. Responses of THP-1 Derived Macrophages to CaP Granules

**Figure 2:**
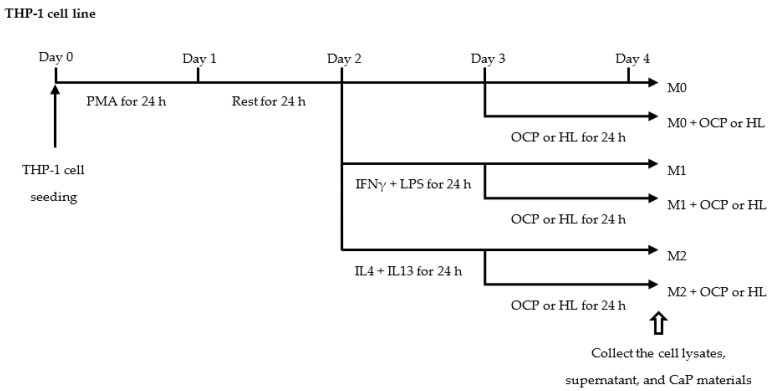
THP-1 cell treatment flowchart. THP-1 cells were differentiated into M0 macrophages by 24 h incubation with 100 ng/mL PMA. THP-1 derived macrophages were polarized into M1 macrophages by incubation with 20 ng/mL IFNγ and 10 pg/mL of LPS for 24 h. THP-1 derived macrophages were polarized into M2 macrophages by incubation with 20 ng/mL of recombinant human IL4 and 20 ng/mL of recombinant human IL13. Three phenotypes of THP-1 derived macrophages or RAW 264 macrophages were treated with OCP or HL granules for 24 h.

**Figure 3:**
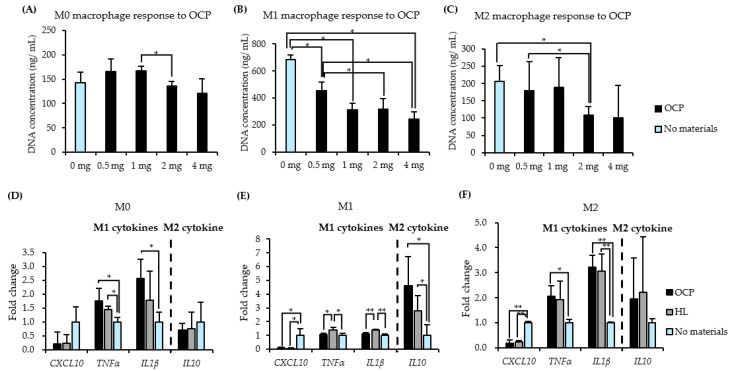
Responses of THP-1 derived macrophages to CaP granules. (**A**–**C**) DNA content of THP-1-derived M0, M1, and M2 macrophages treated with different OCP granules doses (0–4 mg). (*n* = 3) (**D**–**F**) Expression of M1 or M2 macrophage-related cytokines on THP-1-derived M0, M1, and M2 macrophages with or without the 24 h-treatment of 0.5 mg OCP or HL granules. (*n* = 3–4) ** *p* < 0.01, * *p* < 0.05.

Human THP-1 cells were differentiated and then polarized to M1 and M2 macrophages, as shown in Figure 2. The proliferation activity of M0, M1, and M2 macrophages was assessed to optimize the dose of OCP granules for macrophage treatment. Quantification of DNA content (Figure 3B) showed that OCP resulted in a significant decrease in M1 macrophage proliferation compared to that in the absence of OCP. In terms of M0 and M2 macrophages, OCP (0.5 mg) did not induce an evident decrease in DNA content compared to the No materials groups (Figure 3A,C). As a result, a 0.5-mg dose of CaP granules was selected to treat THP-1 derived macrophages.

Figure 3D–F present the expression of M1 and M2-related cytokines in the mRNA level of THP-1-derived M0, M1, and M2 macrophages treated with 0.5 mg OCP or HL for 24 h. The culturing of M0 or M2 macrophages with CaP granules induced an up-regulation of pro-inflammatory genes (*TNFα* and *IL1β*) compared with that of the No materials group (Figure 3D,F). Significant differences were observed in the expression of *TNFα* and *IL1β* in M0 and M2 macrophages in OCP and HL groups compared to the No materials groups (Figure 3D,F), which implies that CaP granules increased the inflammatory response of M0 and M2 macrophages during the 24-h incubation period. *IL10* expression in M1 macrophages was up-regulated in both CaP granules (Figure 3E). HL granules further up-regulated the expression of pro-inflammatory genes (*TNFα* and *IL1β*) compared to those in the No materials and OCP groups. OCP granules suppressed pro-inflammatory gene expression but up-regulated the anti-inflammatory gene (*IL10*) expression in M1 macrophages compared with those in the HL group, which suggests that OCP granules represent a relatively weaker stimulation of inflammatory response in the classically activated M1 macrophages compared to the HL granules.

### 2.3. Responses of RAW 264 Cells to CaP granules

**Figure 4:**
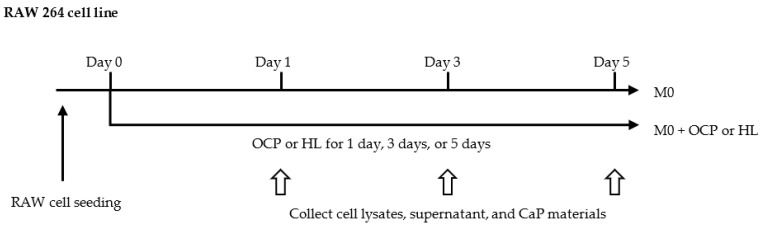
RAW 264 cell treatment flowchart. After seeding RAW 264 cells for 24 h, RAW cells were treated with OCP or HL granules for 1, 3, and 5 days. At each time point, the cell lysates, supernatant, and the soaked CaP granules were collected.

**Figure 5:**
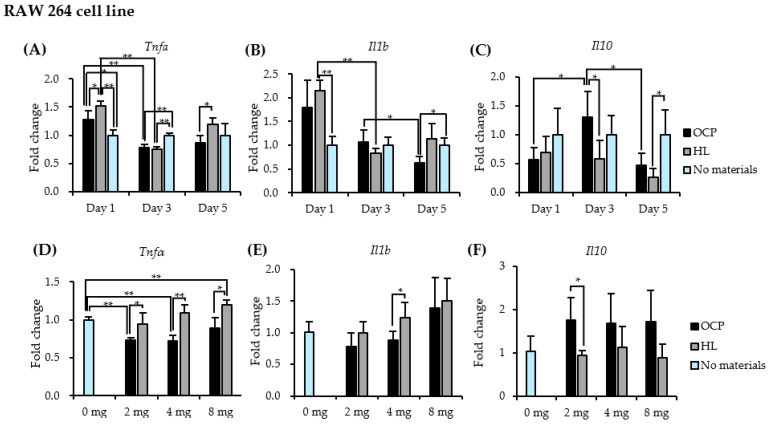
Responses of RAW cells to calcium phosphate granules. (**A**–**C**) The expression of *Tnfα*, *Il1β*, and *Il10* on RAW 264 macrophages with or without the treatment of 4.0 mg OCP or HL granules for 1, 3, and 5 days. (*n* = 4) (**D**–**F**) The expression of *Tnfα*, *Il1β*, and *Il10* on RAW 264 macrophages with different doses of OCP or HL granules (2, 4, and 8 mg) for 3 days. (*n* = 4) ** *p* < 0.01, * *p* < 0.05.

RAW 264 cells were treated with CaP granules for 5 days, as shown in Figure 4. Figure 5 shows the macrophage phenotype pattern in the mRNA level of RAW 264 cells treated with 4.0 mg OCP or HL granules for 5 days. As shown in Figure 5A–C, the expression of pro-inflammatory genes (*Tnfα* and *Il1β*) was up-regulated by both CaP granules on day 1 but decreased after day 3. Compared with the HL group, the expression of *Tnfα* was significantly lower in the OCP group on days 1 and 5. In the OCP group, the expression of the anti-inflammatory gene *Il10* was elevated on day 3 and then decreased on day 5. Moreover, the expression of *Il10* was significantly higher in the OCP group than in the HL group on day 3.

The dose response of CaP granules on the gene expression of M1 and M2 macrophage-related cytokines was investigated by incubating RAW cells with different doses (2, 4, and 8 mg) of OCP or HL granules for 3 days (Figure 5D–F). The mean expression of M1-related cytokines (*Tnfα* and *Il1β*) slightly increased with an increase in the dose of OCP and HL (Figure 3D,E). The expression of *Tnfα* in the OCP group was lower than that in the HL group at doses of 4 and 8 mg. The expression of *Il1β* in the OCP group was lower than that in the HL group at a dose of 4 mg. The difference in the mean values between the OCP and HL groups decreased with the increasing dose. The mean expression of *Il10* in the OCP group was higher than that in the HL group, regardless of the dose (Figure 5F). *Il10* expression was not influenced by the dose of CaP granules.

### 2.4. Changes in the Ionic Microenvironment in the Macrophage Medium in the Presence of CaP Granules

**Figure 6:**
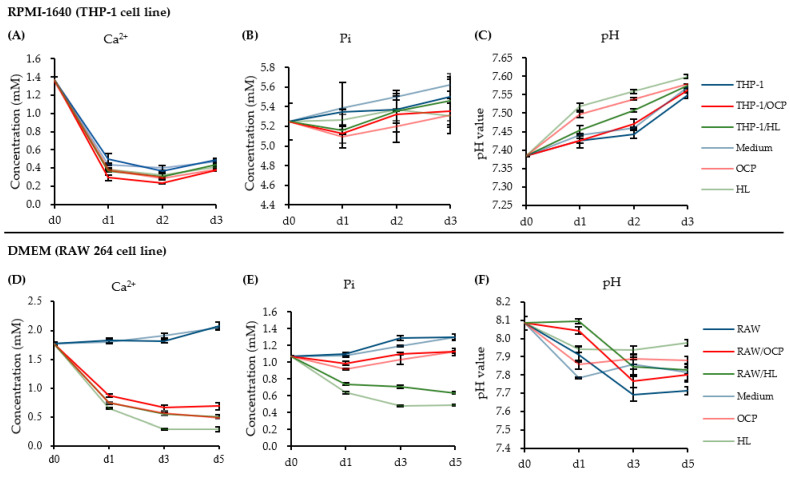
Measurement of the concentration of Ca^2+^, Pi, and pH in the RPMI-1640 medium and DMEM. (**A**–**C**) Ca^2+^ concentration, Pi concentration, and pH value of THP-1 derived M0 macrophages induced by OCP or HL granules in RPMI-1640 medium for 3 days (*n* = 3). (**D**–**F**) Ca^2+^ concentration, Pi concentration, and pH value of RAW 264 cells induced by OCP or HL granules in DMEM for 5 days (*n* = 4).

The effect of CaP granules on macrophages could be related to the changes in the ionic microenvironment, as CaP materials did not have direct contact with macrophages in the cell culture insert. We focused on the change in ionic behavior, where OCP and HL were present. The ion concentrations of Ca^2+^ and Pi in the medium with and without CaP granules were measured over 3 days (incubated with THP-1-derived M0 macrophages, as shown in Figure 6A,B) or over 5 days (incubated with RAW cells, as shown in Figure 6D,E). 

Roswell Park Memorial Institute (RPMI)-1640 medium for THP-1 cells has unique characteristics, with higher Pi and lower Ca^2+^ concentrations compared to the Dulbecco’s modified eagle medium (DMEM). The results of incubation with THP-1-derived macrophages showed that Ca^2+^ and Pi ion concentrations of the CaP granules immersed in an RPMI-1640 medium slightly decreased after 1 day compared with the THP-1 group, but were gradually closer to the THP-1 group after 3 days. Ca^2+^ concentration in the THP-1/HL group remained lower than that in the THP-1 group, but higher than that in the THP-1/OCP group during the 3-day incubation period. The pH value gradually increased during the 3-day incubation period, with no significant difference (Figure 6C). 

The results of incubation with RAW cells suggested that the Pi ion concentration in the DMEM in the presence of HL granules sharply decreased after 1 day and remained lower than that of other groups for up to 5 days. The Pi ion concentration in the RAW/OCP group remained lower than that in the RAW group, but higher than that in the RAW/HL group during the 5-day incubation period. The Ca^2+^ concentration of the medium in the presence of both CaP granules sharply decreased after 1 day and remained lower than that of the RAW group for up to 5 days. During the 5-day incubation period, the Ca^2+^ concentration in the RAW/OCP group remained lower than that in the RAW group, but was slightly higher than that in the RAW/HL group. The pH value of the medium in the presence of CaP granules remained slightly higher and gradually approached that of the RAW group (Figure 6F).

### 2.5. Dose-Response of CaP Granules in an Ionic Microenvironment

**Figure 7:**
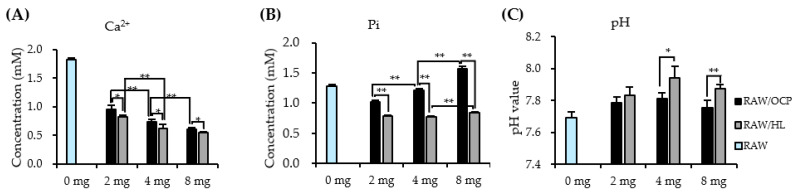
Dose response of CaP materials on the changes in the concentration of Ca^2+^ (**A**), Pi (**B**), and pH (**C**) in the macrophage cultured DMEM. (*n* = 4). The data of the ionic concentration and pH value of RAW (0 mg) group is used from Figure 6. ** *p* < 0.01, * *p* < 0.05. The RAW (0 mg) group is statistically significantly different from other groups in terms of Ca^2+^ concentration (all material groups), Pi concentration (all material groups), and pH value (RAW/OCP 2, 4 mg; RAW/HL 2, 4, 8 mg).

The ion concentration and pH value in the macrophage cultured DMEM were measured by culturing RAW cells with different doses (0, 2, 4, and 8 mg) of OCP or HL granules for 3 days (Figure 7). 

Ca^2+^ concentration decreased as the dose of both CaP granules increased in a dose-dependent manner. Moreover, the Ca^2+^ concentration was always higher in the RAW/OCP group than in the HL group. The Pi ion concentration increased as the dose of OCP granules increased in a dose-dependent manner. The Pi concentration in the RAW/OCP group was always higher than that in the RAW/HL group under various dose conditions. The change in the pH value influenced by the different dose conditions was small in both CaP groups. However, the pH value in the RAW/OCP group was always lower than that in the RAW/HL group under different dose conditions.

### 2.6. Analysis of the Degree of Supersaturation (DS) in DMEM

To clarify the effect of the microenvironment in the media on the polarization of macrophages, we calculated the DS of CaPs (Table 1). Since the RPMI-1640 medium used for THP-1 cell culture has a very high Pi concentration compared to the human serum Pi concentration (approximately 1.12 to 1.45 mM) [24], we adopted DMEM, which is closer to the physiological environment, to calculate the DS values. DS values equal to 1.0, <1.0, and >1.0, represent the conditions of saturation, undersaturation, and supersaturation, respectively.

The DS with respect to HA was the highest (from 1.79 × 10^9^ to 3.28 × 10^14^), followed by the DS with respect to OCP (from 4.54 × 10^4^ to 2.13 × 10^10^) in all groups. These results indicate that HA was more likely to precipitate than OCP in the medium under all conditions.

The DS with respect to HA was higher in the RAW cell-only medium than in the medium in which RAW cells were cultured in the presence of CaP during all periods of culture. Comparing the RAW/OCP group with the RAW/HL group, the DS with respect to HA was similar after 1 day of culture. However, after 3 and 5 days of culture, the DS of the RAW/OCP group was higher than that of the RAW/HL group. This suggests that RAW/OCP may be more prone to precipitate HA and promote OCP-HA conversion than the RAW/HL group, depending on cell culture time.

### 2.7. Characterization of the Spectroscopic Features of CaP Granules

**Figure 8:**
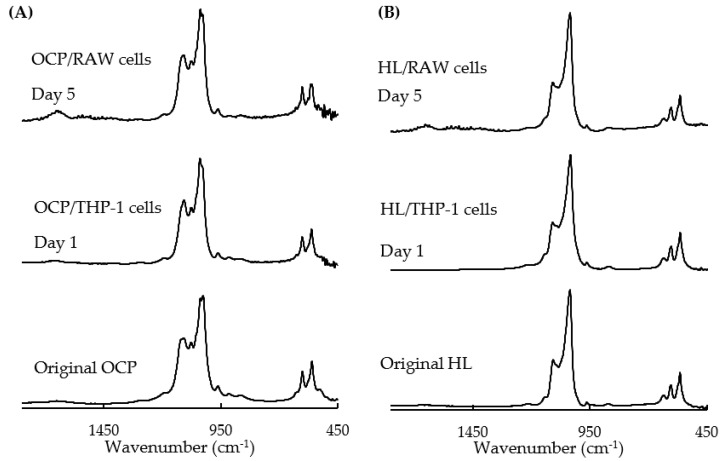
Changes in the FTIR spectra of (**A**) OCP granules or (**B**) HL granules before and after immersion in RAW 264 cell culture medium for 5 days and THP-1-derived macrophage culture medium for 1 day.

Figure 8 shows the Fourier transform infrared spectroscopy (FTIR) spectra of OCP (Figure 8A) and HL (Figure 8B) before and after immersion in the macrophage culture medium. The characteristic peaks for OCP at 1030 and 1078 cm^−1^ became obscured with time in the FTIR spectra of OCP/RAW cells and OCP/THP-1-derived macrophage cells. This suggests that the OCP tended to convert to an apatite structure after incubation with the macrophage culture medium.

## 3. Discussion

Numerous studies have reported macrophage polarization patterns of various CaP biomaterials, but the mechanisms underlying the crystal phases of CaP-based immune responses remain unknown. In the present study, two synthetic CaP materials (OCP and HL) with similar crystal morphologies and granule sizes but different crystal phases were examined. We successfully polarized THP-1-derived M0 macrophages into M1 and M2 macrophages, which were identified by M1 (*TNFα*, *IL1β*, *CXCL10*) and M2 (*IL10*)-related cytokines (Figure 1). Activated macrophages, particularly pro-inflammatory M1 macrophages, showed low cell viability following treatment with OCP granules (Figure 3A–C). Stimulation of both CaP granules on M0 and M2 macrophages suggested that CaP materials induced these two types of macrophages to produce a pro-inflammatory phenotype by increasing the expression of genes of M1-related cytokines (Figure 3D,F). Changes in ion concentration and pH in the media altered the dissolution and precipitation behaviors of CaP and may affect the cellular responses of THP-1 and RAW 264 cells in the presence of OCP and HL granules (Figure 6 and Figure 7). The FTIR spectra suggested that OCP tended to convert to HA depending on the cell culture period (Figure 8). 

In terms of the capacity to stimulate the immune response, OCP considerably promoted the recruitment of macrophages and the release of IL6, which is a pro-inflammatory cytokine. This phenomenon was proven by an in vivo bone defect model and an in vitro macrophage migration assay [18]. This suggests that an inflammatory microenvironment induced by the immune system is beneficial to the osteogenic capacity of biomaterials [10]. In the present study, it was demonstrated that CaP materials can activate the inflammatory response of macrophages and stimulate the pro-inflammatory phenotype. Furthermore, CaP materials seemed to induce M2 macrophage phenotype repolarization to the M1 phenotype by increasing the expression of *TNFα* and *IL1β* genes (Figure 3F). An excessive switch to the M2 phenotype results in scar formation or an extended healing process [25]. In terms of the pro-inflammatory M1 phenotype, OCP and HL granules modulated M1 phenotype polarity in different directions (Figure 3E). Compared with OCP granules, HL granules enhanced the expression of pro-inflammatory genes. Studies have reported that the prolonged M1 polarization promotes the release of fibrogenesis-related cytokines (TNFα, TGFβ1, TGFβ3) via the M2 macrophage phenotype and tends to form fibrous capsulation [26,27]. TNFα and IL1β are pro-inflammatory cytokines that induce osteoclastic activity and result in bone resorption. Compared with OCP, the excessive inflammatory cytokines induced by HL granules may delay the bone-healing process. 

RAW 264 macrophages were treated with CaP materials for a longer period (5 days) to evaluate the effect of CaP materials on macrophage phenotype and functional status. The expression of pro-inflammatory genes in macrophages increased after encountering both CaP materials (day 1), but decreased over time (days 3 and 5) (Figure 5A,B). This agrees with the results from THP-1 cell-derived M0 macrophage model studies, which suggest that inflammation occurs after the initial identification of materials but is alleviated over time [28,29]. Our findings also demonstrated that OCP induced the expression of the anti-inflammatory gene (*IL10*) up to 3 days after treatment with CaP materials, which indicated M2 macrophage polarization (Figure 5C). In contrast, HL displayed less expression of the *IL10* gene from day 3 to day 5 and increased expression of *TNFα* on days 1 and 5. OCP induced different distribution patterns of the M1 and M2 macrophages and pronounced the promotion of M2 macrophages compared to HL. This indicated that the immunomodulation capacity of OCP might have a shorter inflammatory stage and trigger the macrophage phenotype switch from a pro-inflammatory phenotype to an anti-inflammatory phenotype.

The immunomodulation capacity of different CaP materials might be related to the microstructure of CaP materials, such as surface topography, particle size, and material form [30,31,32]. In the present study, inorganic ion dissolution driven by materials could be one of the factors that affected macrophage behavior. Our results suggest that OCP increased Pi ion concentration when RAW cells were cultured in DMEM (Figure 6 and Figure 7). Previous studies revealed that 2.5 mM Pi, which is higher than the serum Pi concentration, promotes the expression of *Arg1*, an M2 marker, and induced an M2 macrophage phenotype [33]. In the present study, Pi concentrations of RAW/OCP and RAW/HL groups were approximately 1.0 mM and 0.7 mM, respectively, and close to that of serum in vivo. Regarding Ca^2+^, it has been reported that RAW cell culture using the supernatant immersed in β-TCP up-regulates the expression of anti-inflammatory genes *IL10* and *IL1ra* via the calcium-sensing receptor (CaSR) pathway [31]. We have previously reported that OCP promoted the migration of J774.1 cells (mouse macrophages) at a 0.6 mM Ca^2+^ concentration [18]. In the present study, the Ca^2+^ concentration in the DMEM tended to decrease in both the RAW/OCP and RAW/HL groups rather than in the RAW (No materials) group, suggesting that this difference may have affected the polarization of the macrophages. Furthermore, it has been reported that pH affects macrophages polarization [34]. Acidic environments (pH = 6.6) tend to polarize macrophages into M2 macrophages, whereas alkaline environments (pH = 8.2) tend to polarize them into M1 [34]. In the present study, the pH of the RAW/OCP and RAW/HL groups was slightly higher (pH = 7.8–8.0) than that of the RAW group, which may have promoted the polarization to M1 macrophages. Since a previous study has shown that the pH of the OCP surface could be controlled depending on the advancement of its hydrolysis into HA [35], the polarization of macrophages may be affected by OCP hydrolysis if the RAW cells were cultured near the granules instead of in the transwell.

Calculating the DS of calcium phosphate from Ca^2+^ and Pi concentrations and pH in DMEM, DS with respect to HA at 3 and 5 days of RAW cell culture was higher in the RAW/OCP group than in the RAW/HL group, suggesting that OCP granules in the DMEM are in a microenvironment that promotes HA precipitation and conversion of OCP to HA with increasing culture days. FTIR analysis showed that OCP tended to convert to HL during RAW 264 cell culture (Figure 8). OCP also modulated the expression of *TNFα* and *IL1β* genes in a dose-dependent manner (Figure 5D,E). These findings suggest that the degree of the inflammatory phenotype may be closely dependent on the local ionic microenvironment.

Recently, the elucidation of the effect of CaP materials on the crosstalk between bone-related cells and immune cells has been the focus of studies [28,32,36]. In this regard, the observed promotion of *IL10* expression was significant (Figure 5C,F). IL10 is an anti-inflammatory cytokine and a critical factor that facilitates bone regeneration [37]. This result suggests that macrophage polarization around crystals may stimulate OCP osteoconductivity.

## 4. Materials and Methods

### 4.1. Synthesis of CaP Granules

OCP and HL were obtained using a wet precipitation method at 65 °C, as previously described [8]. HL was obtained through the conversion of OCP slurry with constant stirring at 65 °C for 48 h. Granules of OCP and HL at a size of 300–500 µm were prepared by sieving the materials between 32 and 48 mesh. The sieved granules were sterilized at 120 °C for 2 h.

### 4.2. Cell Differentiation, Polarization, and Treatment

In this study, two cell models were used: human monocytes (THP-1 cells) and mouse macrophages (RAW 264 cells). THP-1 cells were purchased from the Cell Resource Center for Biomedical Research, Institute of Development, Aging and Cancer, Tohoku University (Sendai, Japan). THP-1 cells were maintained in an RPMI-1640 medium (Gibco, Waltham, MA, USA) containing 10% heat-inactivated fetal bovine serum (FBS, Gibco, Waltham, MA, USA), 1% penicillin/streptomycin mixed solution (Nacalai Tesque Inc., Kyoto, Japan), supplemented with 10 mM HEPES (Gibco, Waltham, MA, USA), 1 mM sodium pyruvate (Gibco, Waltham, MA, USA), and 0.11 mM β-mercaptoethanol (Gibco, Waltham, MA, USA) at 37 °C in a 5% CO_2_ incubator until the assay was performed. THP-1 monocytes were differentiated into M0 macrophages by 24-h incubation with 100 ng/mL PMA (Sigma, Munich, Germany) followed by 24-h incubation in the RPMI-1640 complete medium. Macrophages were polarized into M1 macrophages, which were classified into pro-inflammatory macrophages, by incubation with 20 ng/mL IFNγ (R&D systems, Minneapolis, MN, USA) and 10 pg/mL of LPS (*Escherichia coli* O111: B4, Sigma, Munich, Germany) for 24 h. Macrophages were polarized into M2 macrophages, which are anti-inflammatory macrophages, by incubation with 20 ng/mL of recombinant human IL4 (R&D Systems, Minneapolis, MN, USA) and 20 ng/mL of recombinant human IL13 (R&D Systems, Minneapolis, MN, USA) for 24 h (Figure 2).

A cell culture insert system was used to treat macrophages with OCP or HL granules. The proliferative activity of the macrophages treated with OCP was determined by the quantification of DNA content using a Quant-iT^TM^ PicoGreen^®^ dsDNA Reagent and Kits (Invitrogen, Waltham, MA, USA). Briefly, THP-1-derived M0, M1, and M2 macrophages were treated with 0, 0.5, 1, 2, or 4 mg of OCP granules, which were placed in a transwell (8.0 µm pore size, FALCON, New York, NY, USA) in a 24-well plate. After incubation for 24 h, the amount of double-stranded DNA in the cell lysate was assessed by a fluorometric assay using PicoGreen. THP-1-derived M0, M1, or M2 macrophages were treated with 0.5 mg OCP or HL granules for 24 h or 3 days in an FBS-free RPMI-1640 medium.

RAW 264 cells were purchased from Riken Cell Bank (Tsukuba, Japan). RAW 264 cells were maintained in DMEM (Wako, Japan) containing 10% heat-inactivated fetal bovine serum (FBS) (Gibco, Waltham, MA, USA), and 1% penicillin/streptomycin solution (Nacalai Tesque Inc., Kyoto, Japan) at 37 °C in a 5% CO_2_ incubator until the assay was performed.

RAW 264 cells were treated with 4.0 mg OCP or HL granules for 5 days in the DMEM complete medium (Figure 4). The dose response of CaP materials was evaluated by treating RAW 264 cells with 2.0, 4.0, or 8.0 mg OCP or HL granules for 3 days in DMEM.

At the respective time points, cell lysates, the supernatant of the cultured medium, and the soaked granules were harvested.

### 4.3. Evaluation of the Expression of M1 and M2 Macrophage-Related Genes by qPCR

Total RNA was extracted using a TRIzol reagent (Life Technologies, Carlsbad, CA, USA) following the manufacturer’s protocol. Reverse transcription was performed using ReverTra Ace^®^ qPCR RT Master Mix with gDNA remover (TOYOBO Life Science, Osaka, Japan). Optimal oligonucleotide primers and TaqMan probes were designed using ProbeFinder software (Roche, Basel, Switzerland). The mRNA expression levels of M1 macrophage-related genes (*TNFα*, *IL1β*, *CXCL10*), M2 macrophage-related gene (*IL10*), and housekeeping gene (*GAPDH*) were determined by real-time TaqMan polymerase chain reaction (RT-PCR) analysis. The sequences of the PCR primers and universal probes are listed in Table 2. Quantitative RT-PCR was conducted in a 20 μL reaction volume with 1× FastStart Essential DNA Probe Master (Roche, Basel, Switzerland), 500 nM of forward and reverse primers each, 200 nM Universal ProbeLibrary probe, and the cDNA template. Cycling conditions were as follows: preincubation at 95 °C for 600 s followed by 2-step amplification for 45 cycles at 95 °C for 10 s and 60 °C for 30 s. RT-PCR was performed using a LightCycler^®^ 96 system (Roche Applied Science, Mannheim, Germany). The relative expression levels of the target mRNAs were normalized against the *GAPDH* gene. The No materials groups at each time point were used as the calibrator samples.

### 4.4. Measurement of Changes in the Ionic Microenvironment in the CaP Granule-Treated Macrophage Culture Medium

The supernatant of the macrophage medium treated with CaP was harvested at the respective time points. Changes in the concentration of Ca^2+^ and Pi ions in the medium were quantitatively determined using calcium E and Phosphor C tests (Wako Pure Chemical Industries, Osaka, Japan), respectively. The pH of the culture medium was measured using a pH electrode (Horiba Ltd., Kyoto, Japan).

### 4.5. Determination of the DS in the DMEM Immersed with OCP or HL Granules

The DS collected in the DMEM immersed in OCP or HL granules was calculated to estimate the solubility with respect to HA, OCP, and calcium hydrogenphosphate dihydrate (DCPD) in the media. The DS can be expressed by dividing the ionic product by the solubility product of the objective calcium phosphate phases. The ionic activity products for calcium phosphate were calculated using the analytical results of [Ca^2+^], [Mg^2+^], [Na^+^], [K^+^], [Pi], [Cl^−^], and [F^−^], as well as the pH value, in conjunction with the 3 mass balance equations for [Ca^2+^], [Pi], and [Mg^2+^], according to previous reports [38,39,40]. In the present study, the pH and concentration of Ca^2+^ and Pi obtained using chemical analyses (Figure 6) were used. The ion pairs considered were CaH_2_PO_4_^+^, CaHPO_4_^0^, MgHPO_4_^0^, CaHCO^3+^, and MgHCO^3+^. The DS was estimated in terms of the mean ionic activity products with respect to HA, OCP, and DCPD. Na^+^ concentration in the DMEM was assumed to be 155.3 mM from calculating the sodium included in the medium. Mg^2+^ and F^−^ are assumed to be approximately zero. The solubility product constants used were 7.36 × 10^−60^ (mol/L)^9^ for HA [41], 2.51 × 10^−49^ (mol/L)^8^ for OCP [42], and 2.77 × 10^−7^ (mol/L)^2^ for DCPD [43] at 37 °C.

### 4.6. FTIR Analysis of OCP and HL after Immersion in the Macrophage Medium

CaP granules incubated for 1 day (with THP-1 derived macrophages) or 5 days (with RAW cells) at 37 °C in a 5% CO_2_ environment were characterized by FTIR (IR-6300; JASCO Corporation, Tokyo, Japan) with the dilution in potassium bromide over a range of 4000–400 cm^−1^ and a resolution of 4 cm^−1^.

### 4.7. Statistical Analysis

Results are expressed as mean ± standard deviation (SD). Student’s *t*-test was used to determine statistical differences between the two groups. For all comparisons, a *p*-value < 0.05, was considered statistically significant.

## 5. Conclusions

In the present study, we demonstrated that CaP materials regulate macrophage polarization patterns. OCP provides a beneficial immunomicroenvironment for macrophages to activate the inflammatory response and quickly switch to an anti-inflammatory phenotype. This response raised by OCP seems to be related to changes in the ion concentration and pH value in the microenvironment, depending on the crystal phases of the CaP materials. Further studies will clarify the mechanisms triggered by the crystal phase of CaP materials to modulate the immune response and promote bone regeneration.

## Figures and Tables

**Table 1:** Solution composition and degree of supersaturation (DS) of macrophage culture medium before and after the immersion of OCP or HL granules.

	Period (Days)	Ca^2+^ (mM)	Pi (mM)	pH	DS at pH (Each) and 37 °C		
					HA	OCP	DCPD
DMEM	0	1.78	1.07	8.08	3.28 × 10^14^	4.54 × 10^4^	6.72 × 10^−1^
RAW cells	1	1.82	1.10	7.91	7.84 × 10^13^	2.31 × 10^4^	6.90 × 10^−1^
RAW/OCP	1	0.88	0.99	8.04	6.13 × 10^12^	2.03 × 10^3^	3.18 × 10^−1^
RAW/HL	1	0.75	0.74	8.10	2.03 × 10^12^	6.12 × 10^2^	2.07 × 10^−1^
OCP	1	0.75	0.92	7.86	3.78 × 10^11^	3.46 × 10^2^	2.48 × 10^−1^
HL	1	0.65	0.64	7.94	1.50 × 10^11^	1.06 × 10^2^	1.54 × 10^−1^
RAW cells	3	1.82	1.28	7.69	1.44 × 10^13^	1.16 × 10^4^	7.68 × 10^−1^
RAW/OCP	3	0.66	1.10	7.77	1.38 × 10^11^	2.19 × 10^2^	2.56 × 10^−1^
RAW/HL	3	0.56	0.71	7.85	3.90 × 10^10^	4.96 × 10^1^	1.45 × 10^−1^
OCP	3	0.57	1.03	7.89	1.82 × 10^11^	1.91 × 10^2^	2.13 × 10^−1^
HL	3	0.30	0.48	7.94	1.37 × 10^11^	2.13 × 10^0^	5.43 × 10^−2^
RAW cells	5	2.08	1.30	7.72	3.52 × 10^13^	2.26 × 10^4^	8.87 × 10^−1^
RAW/OCP	5	0.69	1.12	7.80	2.56 × 10^11^	3.30 × 10^2^	2.74 × 10^−1^
RAW/HL	5	0.51	0.64	7.83	1.44 × 10^10^	2.19 × 10^1^	1.17 × 10^−1^
OCP	5	0.48	1.12	7.88	9.04 × 10^10^	1.18 × 10^2^	1.95 × 10^−1^
HL	5	0.30	0.49	7.98	1.79 × 10^9^	2.41 × 10^0^	5.39 × 10^−2^

The Ca^2+^ concentration, Pi concentration, and pH data for each group are shown in Figure 6D–F. The DS in each group was calculated according to the ionic concentration and pH (each).

**Table 2:** Primers and probes for RT-PCR analyses in this study.

Species	Gene	PCR Primers (5′-3′)	Universal Probe
Human	*TNFα*	Forward: agcccatgttgtagcaaacc	#79
		Reverse: tctcagctccacgccatt	
	*IL1β*	Forward: agccaggacagtcagctctc	#23
		Reverse: agaggcctggctcaacaa	
	*CXCL10*	Forward: aagcagttagcaaggaaaggtc	#34
		Reverse: gacatatactccatgtagggaagtga	
	*IL10*	Forward: ttgcctggtcctcctgact	#37
		Reverse: gaagtgggtgcagctgttct	
	*GAPDH*	Forward: ccccggtttctataaattgagc	#63
		Reverse: cttccccatggtgtctgag	
Mouse	*Tnfα*	Forward: ctgtagcccacgtcgtagc	#25
		Reverse: tttgagatccatgccgttg	
	*Il1β*	Forward: agttgacggaccccaaaag	#38
		Reverse: agctggatgctctcatcagg	
	*Il10*	Forward: cagagccacatgctcctaga	#41
		Reverse: tgtccagctggtcctttgtt	
	*Gapdh*	Forward: tgtccgtcgtggatctgac	#80
		Reverse: cctgcttcaccaccttcttg	

## Data Availability

The data were basically provided in the manuscript.
